# Bayesian reanalysis of null results reported in medicine: Strong yet variable evidence for the absence of treatment effects

**DOI:** 10.1371/journal.pone.0195474

**Published:** 2018-04-25

**Authors:** Rink Hoekstra, Rei Monden, Don van Ravenzwaaij, Eric-Jan Wagenmakers

**Affiliations:** 1 University of Groningen, Groningen, The Netherlands; 2 University Medical Center Groningen, Groningen, The Netherlands; 3 University of Amsterdam, Amsterdam, The Netherlands; Janssen Research and Development, UNITED STATES

## Abstract

Efficient medical progress requires that we know when a treatment effect is absent. We considered all 207 Original Articles published in the 2015 volume of the *New England Journal of Medicine* and found that 45 (21.7%) reported a null result for at least one of the primary outcome measures. Unfortunately, standard statistical analyses are unable to quantify the degree to which these null results actually support the null hypothesis. Such quantification is possible, however, by conducting a Bayesian hypothesis test. Here we reanalyzed a subset of 43 null results from 36 articles using a default Bayesian test for contingency tables. This Bayesian reanalysis revealed that, on average, the reported null results provided strong evidence for the absence of an effect. However, the degree of this evidence is variable and cannot be reliably predicted from the *p*-value. For null results, sample size is a better (albeit imperfect) predictor for the strength of evidence in favor of the null hypothesis. Together, our findings suggest that (a) the reported null results generally correspond to strong evidence in favor of the null hypothesis; (b) a Bayesian hypothesis test can provide additional information to assist the interpretation of null results.

## Introduction

Across the medical sciences, null results are of central importance. For both patients and doctors, it is crucial to know that a new treatment does not outperform the current gold standard; that a generic drug is just as effective as an expensive brand-name drug; and that a new surgical procedure does not improve survival rate. Knowing that effects are absent allows the profession to retain existing medical procedures and reallocate its limited resources to the exploration of novel treatments that are potentially effective.

In this manuscript we summarize and reanalyze the null results for primary outcome measures reported in the 2015 volume of the *New England Journal of Medicine* (NEJM). As detailed below, 45 out of 207 Original Articles (21.7%) featured at least one null result. Because of their prominence and impact in the medical literature, null results deserve a detailed and appropriate statistical treatment.

The statistical evaluation of medical hypotheses currently proceeds almost exclusively through the framework of *p*-value null-hypothesis significance testing (NHST) [1,2,3] for a general warning about the use of *p*-values in medical research see [4], and [5], p. 424). This statistical framework is particularly problematic for the assessment of null results, because nonsignificant *p*-values do not quantify evidence in favor of the null hypothesis (e.g., [6, 7, 8, 9, 10]. This important caveat was recently underscored in a report from the *American Statistical Association*: “a relatively large *p*-value does not imply evidence in favor of the null hypothesis” [11], p. 132.

**Fig 1:**
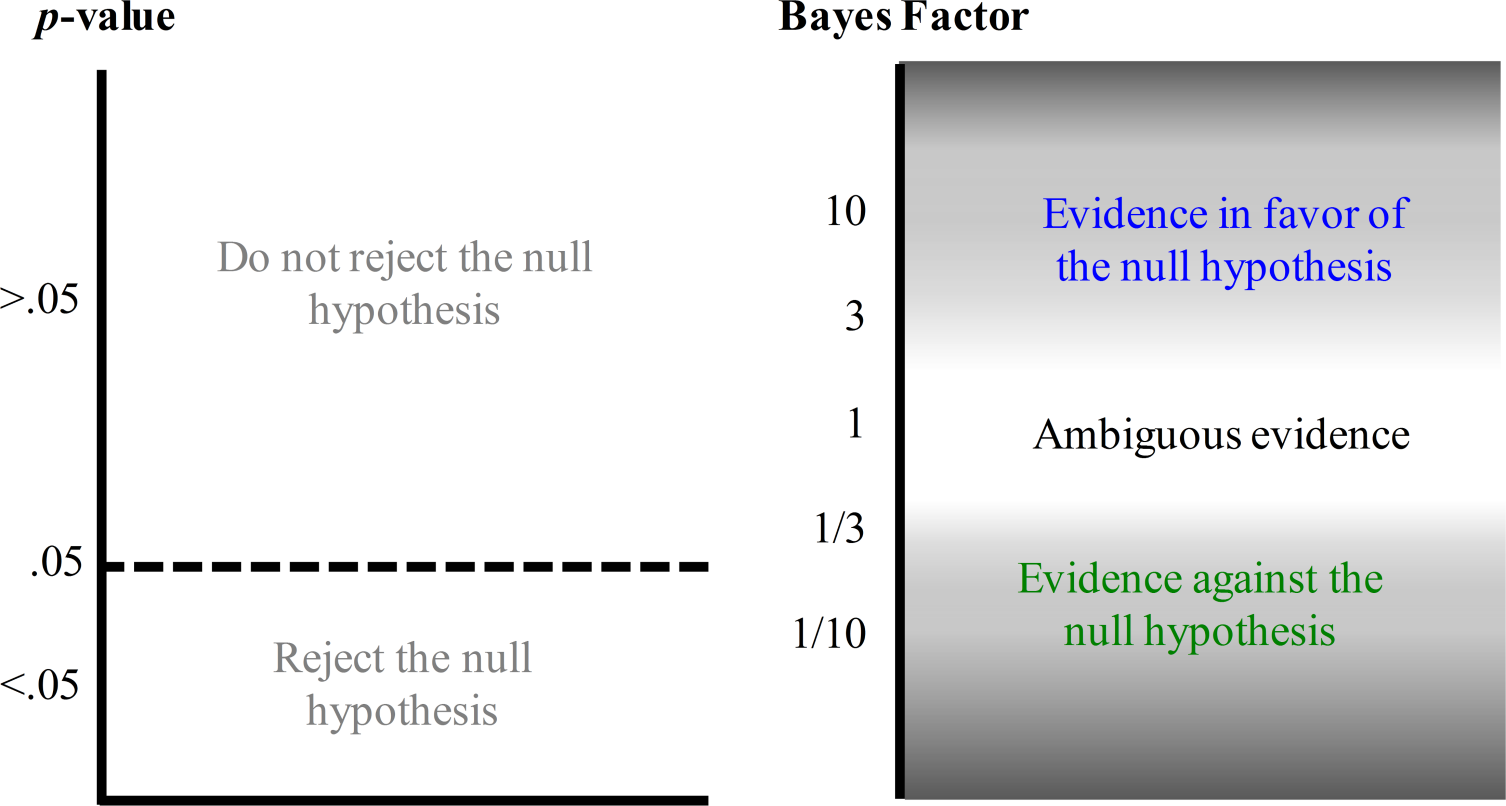
Valid statements based on p-values and Bayes factors. The *p*-value and the Bayes factor allow fundamentally different statements concerning the null hypothesis. The *p*-value can be used to make a discrete decision: reject or retain the null hypothesis. The Bayes factor grades the evidence that the data provide for and against the null hypothesis.

In order to quantify the evidence in favor of the absence of a treatment effect, we adopt the framework of Bayesian statistics and compute the predictive performance of two competing hypotheses: the null hypothesis that states the effect to be absent and the alternative hypothesis that states the effect to be present (e.g., [12, 13]). The resulting balance of predictive performance is known as the *Bayes factor* (e.g., [2, 14, 15, 16, 17]). In contrast to NHST, the Bayes factor allows researchers to quantify evidence in favor of the null hypothesis. For instance, when the Bayes factor BF = 10, the observed data are 10 times more likely under the null hypothesis than under the alternative hypothesis; when BF = 1 the observed data are equally likely under both hypotheses (i.e., the data are perfectly ambiguous and do not favor one hypothesis over the other); and when BF = 1/10 the observed data are 10 times more likely under the alternative hypothesis than under the null hypothesis. Fig 1 highlights the different interpretations that the two statistical frameworks allow.

Computation of the Bayes factor requires the analyst to quantify the expectation about effect size under the alternative hypothesis. In contrast to a classical power analysis, this expectation encompasses a range of different effect sizes, weighted by their prior plausibility. Here we adopt an “objective Bayesian approach” [18] and apply a default test that assigns expectations to effect sizes under the alternative hypothesis such that the test performs well across a wide range of substantively different applications [13].

The conceptual advantage of a Bayesian analysis can be underscored with a simple example. Consider the study by Jolly et al. ([19]; this study is part of the data presented later in this paper) who compared mortality rates of two groups of patients with ST-segment elevation myocardial infarction, one group receiving percutaneous coronary intervention (PCI) and thrombectomy and one group receiving PCI alone. The mortality rate in the PCI-plus-thrombectomy group was 347/5033 (6.89%) and the mortality rate in the PCI-only group was 351/5030 (6.98%). The standard chi-square test yields a *p*-value of .90 (.87 without application of Yates’ continuity correction). Now consider another study by Carrier et al. ([20]; this study is also part of the data presented later in this paper), who examined whether the ability to detect occult cancer is improved by adding computed tomography (CT) to the limited screening practice. The miss rate in the screening-plus-CT group was 5/19 (26.32%) and the miss rate in the screening-only group was 4/14 (28.57%). The standard chi-square test yields a *p*-value of 1 (.89 without application of Yates’ continuity correction).

Both studies fail to reject the null hypothesis and obtain a *p*-value of similar magnitude. Nevertheless, application of the default Bayes factor hypothesis test reveals that the data from the Carrier et al. study, despite the larger *p*-value, provide only weak support for the null hypothesis (i.e., BF = 2.7, implying that the data are not much more likely under the null hypothesis than under the alternative hypothesis) whereas the data from the Jolly et al. study provide compelling support in favor of the null hypothesis (i.e., BF = 77.7). This example illustrates how a Bayes factor hypothesis test can differentiate between data that are almost uninformative and data that are highly diagnostic (e.g., [6]).

In sum, the assessment of medical null effects may benefit from an additional Bayesian analysis. Below we explore the extent to which medical null results reported in the 2015 volume of NEJM actually yield compelling evidence in favor of the null hypothesis when assessed by a default Bayes factor hypothesis test.

## Method

### Sample

We considered all 207 Original Articles published in the 2015 volume of NEJM. This journal was chosen because of its prominence in the field of medicine, and because it publishes papers about a wide range of medical issues. An initial screening identified 45 articles whose abstract contained at least one claim about the absence or non-significance of an effect for a primary outcome measure (21.7%). To facilitate the analysis and the interpretation of the results, we selected a further subset 37 of articles that allowed a simple comparison between proportions, that is, *m* by *k* contingency tables. After eliminating one article whose results were significant upon reanalysis, we obtained a final sample of 36 articles. Several of these articles contained more than one claim of no effect, such that the total number of effects available for reanalysis equaled 43. A detailed description of the articles under consideration and the selection procedure is provided in the supplements.

### Bayes factor reanalysis

Our Bayesian reanalysis was facilitated by the fact that in order to compute Bayes factors for contingency tables, knowledge of the individual cell counts suffices. Bayes factors were calculated using the default test for a comparison of proportions (e.g., [12, 21, 22, 13];) implemented in the statistical software package JASP [23]. JASP is an open-source statistical software package with a graphical user interface, supporting both frequentist and Bayesian analyses (for details see jasp-stats.org). The underlying code base is in R ([24]), and our analyses can also be executed in the BayesFactor package [25], available at https://cran.r-project.org/web/packages/BayesFactor/index.html.

The Bayes factors reported here compare the predictive performance of the null-hypothesis (which assumes the absence of association between rows and columns) against predictive performance of the alternative hypothesis (which assumes the presence of an association). The default Bayes factor specifies that under the alternative hypothesis, every combination of values for the proportions is equally likely a priori. For example, in the case of the 2 by 2 table, the alternative hypothesis specifies two independent uniform distributions for the two rate parameters. In specific applications, such a default, reference-style analysis can be supplemented by substantive knowledge based on earlier experience. With a more informative prior distribution, the alternative hypothesis will make different predictions, and a comparison with the null hypothesis will therefore yield a different Bayes factor. The more informed the prior distribution, the more specific the model predictions, and the more risk the analyst is willing to take. Highly informed prior distributions need to be used with care, as they may exert a dominant effect on the posterior distribution, making it difficult to “recover” once the data suggest that the prior was ill-conceived. With informed prior distributions, it is wise to perform a robustness analysis to examine the extent to which different modeling choices lead to qualitatively different outcomes.

In this article, we prefer the default prior, as it is the most common choice and an informed specification would require an elaborate elicitation process from many different experts. We do not, therefore, view the outcomes of this analysis as definitive, although we believe that the qualitative results (i.e., strong but highly variable evidence in favor of the null) hold across a broad range of prior distributions.

Before proceeding it is important to point out that the Bayes factor quantifies the support provided by the data. For any two models, the posterior odds is obtained by multiplying the Bayes factors by the prior odds. In other words, the Bayes factor allows an assessment of the strength of evidence that is independent from the relative prior plausibility of the models.

## Results

**Fig 2:**
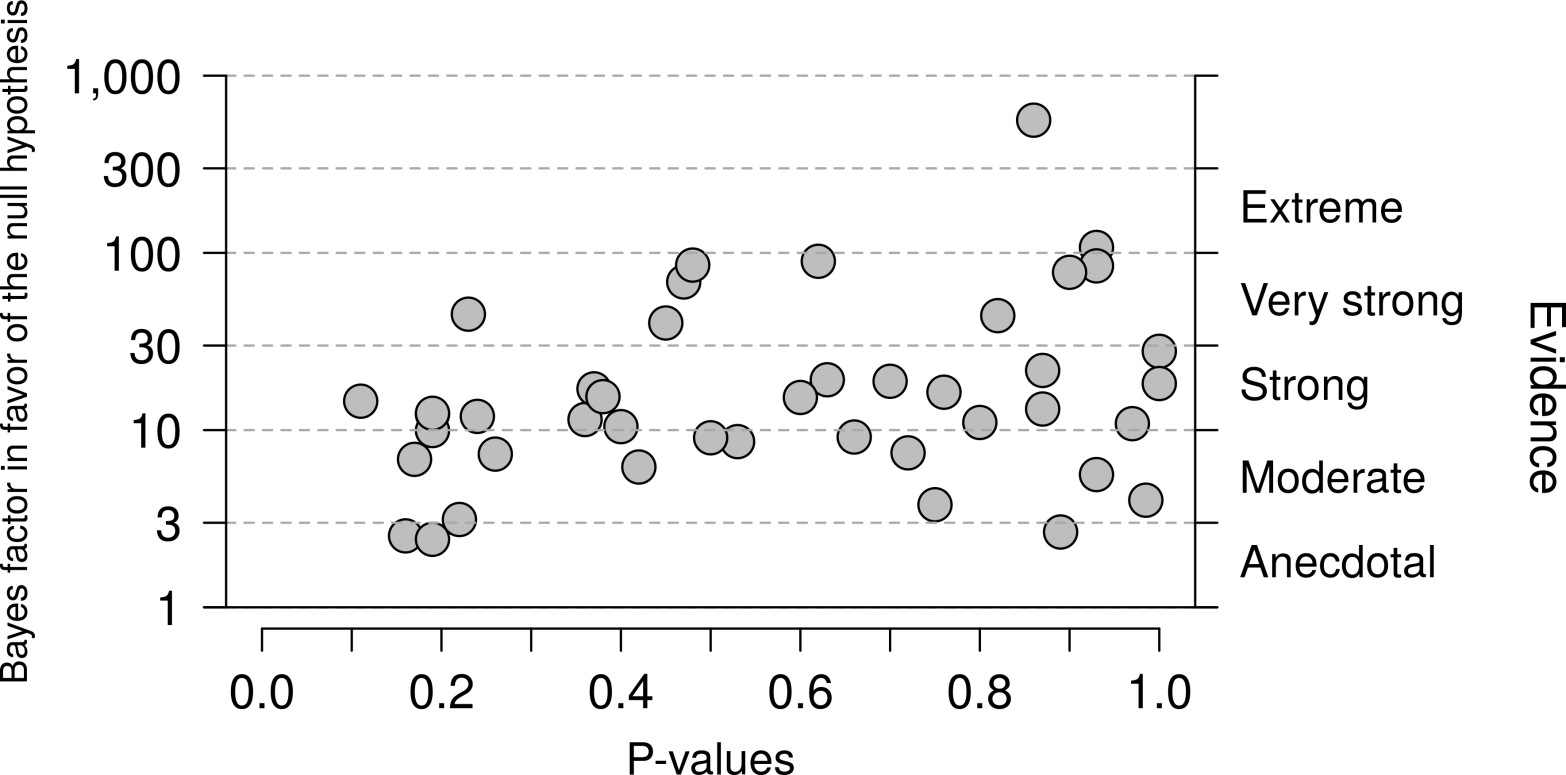
Relation between p-values and Bayes factors. *P*-values and Bayes factors in favor of the null hypothesis for 43 null results from the 2015 volume of NEJM. All Bayes factors indicate support in favor of the null hypothesis, and most Bayes factors do so in a compelling fashion. At the same time, the support in favor of the null hypothesis is highly variable. The *p*-value only explains 8.39% of the variance in the log Bayes factors (r = 0.29).

The main result concerns the default Bayes factors for the 43 null effects reported in the 2015 NEJM volume. Fig 2 shows the Bayes factors (on a logarithmic scale) as a function of the *p*-value. Reassuringly, all Bayes factors are higher than 1, indicating support in favor of the null hypothesis. For ease of interpretation, the right-hand y-axis displays the evidential categories proposed by Jeffreys ([15], Appendix B; the labels have been changed as suggested in [26]). Most Bayes factors provide strong or very strong evidence in favor of the null hypothesis, and only three are weak, anecdotal, or “not worth more than a bare mention” [13].

Fig 2 also shows that the degree of support in favor of the null hypothesis fluctuates considerably from one experiment to the next—the lowest Bayes factors are smaller than 3, whereas the most compelling Bayes factors exceed 100. Moreover, the observed *p*-value is a poor predictor of the Bayes factor. For instance, a *p*-value near 0.9 is almost equally likely to correspond to anecdotal, moderate, strong, or very strong evidence in favor of the null hypothesis. This underscores the usefulness of the Bayes factor over and above the *p*-value.

**Fig 3:**
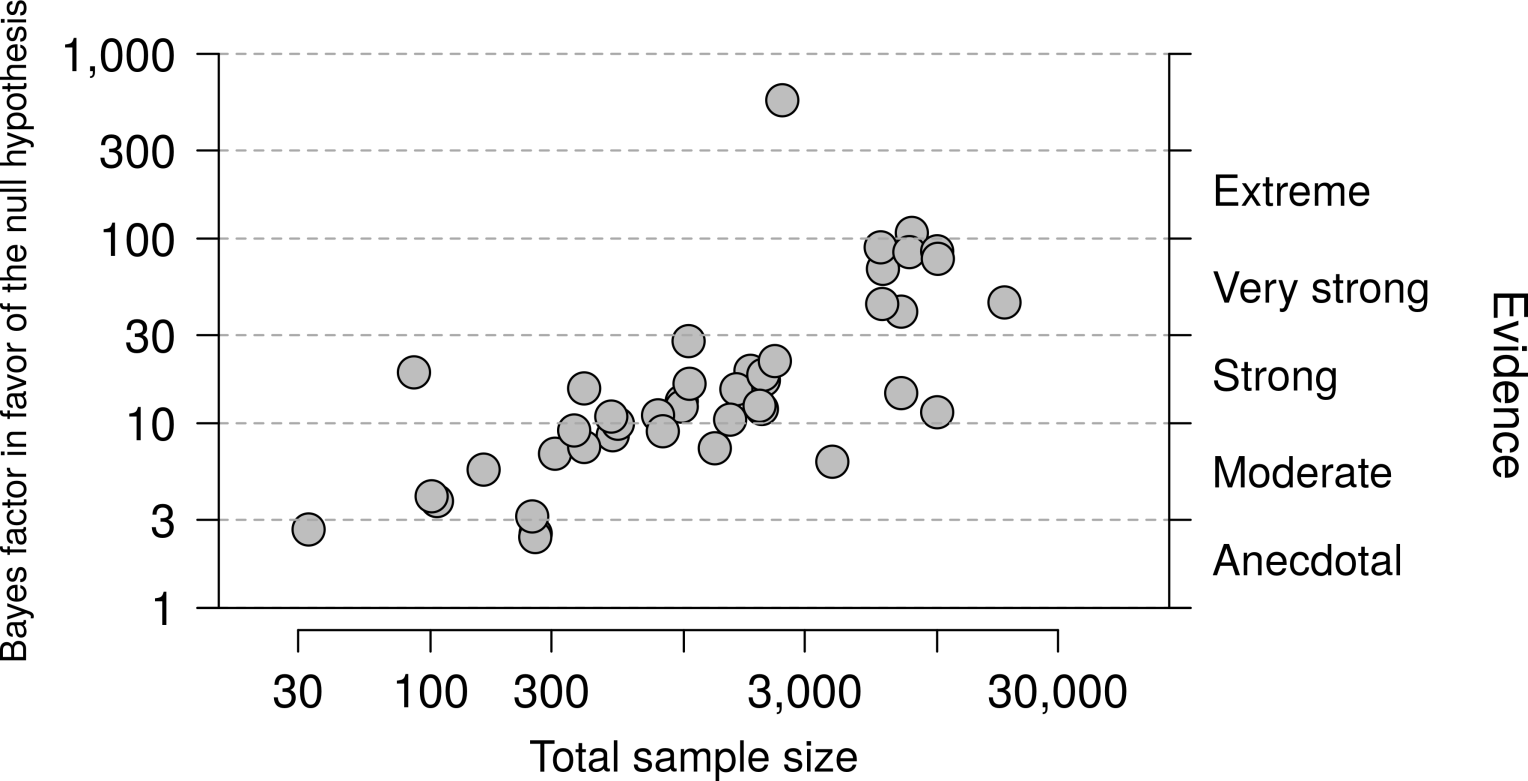
Relation between sample size and Bayes factors. Among the 43 null results from the 2015 volume of NEJM, large samples are more likely to yield compelling evidence in favor of the null hypothesis than small samples (r = 0.72).

One possible determinant of the strength of the Bayes factor is sample size. Fig 3 shows the relation between sample size and Bayes factors (both plotted on a logarithmic scale) and confirms that in a small sample, a nonsignificant result is likely to be nondiagnostic (i.e., the evidence in favor of the null hypothesis is likely to be weak), whereas in a large sample a nonsignificant result is likely to be diagnostic (i.e., the evidence in favor of the null hypothesis is likely to be strong). Nevertheless, sample size alone is not sufficient to gauge the evidence; for instance, even with a total sample size of 10,000 a nonsignificant result may lead to a Bayes factor slightly higher than 10 or a Bayes factor higher than 100.

## Discussion

We applied a default Bayes factor reanalysis to 43 null results published in the 2015 volume of NEJM. Reassuringly, this reanalysis revealed that all null results supported the null hypothesis over the alternative hypothesis, and the overall degree of support was strong. Nevertheless, from experiment to experiment the degree of evidence varied considerably—the smallest Bayes factor was 2.42 (“not worth more than a bare mention”, [15]), whereas the largest Bayes factor was 560.9 (“decisive” or “extreme” evidence, [15]; [26]). Our findings also suggest that for non-significant findings, the degree of evidence in favor of the null hypothesis cannot be predicted from the *p*-value, but can be predicted to some extent from sample size: larger samples sizes are more likely to produce compelling evidence.

Several remarks are in order. First, we were pleasantly surprised that as many as 21.7% of the studied papers reported a null result for at least one of the primary outcome measures, considering the strong confirmation bias that is present in the biomedical literature (e.g., [27]). Second, the outcome of our analysis is, naturally, model-dependent. In our comparison of proportions we adopted a default specification of the alternative hypothesis (e.g., [12,13]). We have made our data available online to encourage additional analyses. Third, we limited ourselves here to the assessment of evidence for what is arguably the most popular statistical analysis across the medical sciences: the comparison between two or more proportions. The canonical example is the comparison between the survival rate in a control group versus that in a treatment group. More complicated experimental designs require the application of different statistical models and associated Bayes factors (e.g., [28]).

Fourth, it may be possible to combine the different ingredients of classical statistics and NHST (e.g., power, confidence intervals, *p*-values, sample sizes, effect sizes) to obtain an intuitive and sensible assessment of the support that the data provide in favor of the null hypothesis. Nevertheless, this concept itself is antithetical to classical statistics. The father of modern classical statistics, Ronald Fisher, famously stated, “the null hypothesis is never proved or established, but is possibly disproved, in the course of experimentation. Every experiment may be said to exist only in order to give the facts a chance of disproving the null hypothesis.” ([29], p. 19). But in medical research, it is often of crucial importance to be able to quantify the support in favor of the absence of an effect. Rather than combining the various ingredients from classical statistics in order to overcome its inherent limitations, we propose that the desired inference can be attained more simply and more directly through the application of Bayesian statistics.

Fifth, one might argue that the point null hypothesis is never exactly true, and as a result its examination is pointless (i.e., a foregone conclusion [30]), whether from a frequentist of Bayesian standpoint. We disagree with this argument. The null hypothesis represents the idealized position of a skeptic, and claims that contradict this position will have a hard time being accepted by the scientific community when the data fail to discredit the skeptic’s position. Moreover, the null hypothesis may often be exactly true. For instance, the effect of homeopathic drugs will be exactly equal to the effect of a placebo alternative, assuming that the study is executed with care. In general, drugs whose active components do not impinge on the relevant biological mechanism will be incapable of outperforming placebo. The most compelling counterargument, in our opinion, is that the null hypothesis is merely a convenient approximation to the true state of nature, in which effects are so small that they cannot be meaningfully studied with realistic samples sizes. As Cornfield [31] put it, “For finite sized samples the probability of rejecting either H_0_ or H_δ_ [with δ representing a very small effect—HMRW] will be nearly equal, and concern about the high probability of rejecting one is equivalent to concern about rejecting the other” (p. 582).

A final point concerns the difference between testing and estimation. Several researchers and institutions (e.g., [32,33,34]) have promoted the notion that confidence intervals should supplement or replace p-values. In practice, researchers who report confidence intervals often focus exclusively on whether or not the interval contains the value specified by the null hypothesis, thereby executing significance testing by stealth. Also, confidence intervals cannot be used to quantify the strength of evidence, which we expect is something that many researchers are interested in. Moreover, confidence intervals share many of the same interpretational problems with *p*-values (e.g., [35,36]), reducing the appeal of supplementing of replacing *p*-values with confidence intervals. In contrast, Bayes factors do not share the *p*-value problems; for instance, Bayes factors allow researchers to quantify the evidence in favor of the null hypothesis, which may be crucial for medical practitioners who want to know whether or not a particular treatment is effective. But Bayes factors are a tool for testing, and if one prefers estimation (for instance because there is only a single plausible hypothesis or model) the credible interval is a useful Bayesian alternative to the frequentist confidence interval.

In general, null results in medicine can have serious practical ramifications. The importance of medical null results is evident from the fact that in 2015, about 1 in every 5 papers we studied reported a null result for one of its primary outcome measures. For such null results, medical professionals need to be able to gauge the evidence in favor of the absence of an effect. Here we showed how this goal can be accomplished by the application of a default Bayes factor test. For many standard analyses, such default Bayes factor tests are now easy to apply ([23,25]). We recommend that researchers who report a null result consider the conclusions that follow from both a classical and a Bayesian perspective.

In sum, an assessment of all 207 Original Articles in the 2015 volume from NEJM revealed that 21.7% reported a null result for one or more of their primary outcome measures. A standard Bayesian reanalysis of 43 null results revealed that the evidence in favor of the null hypothesis was strong on average, but highly variable. Higher sample sizes generally produced stronger evidence. We suggest that by adopting a statistically inclusive approach, medical researchers confronted with a null result can issue a report that is more informative and more appropriate than the one that is currently the norm.

## Supporting information

S1 File Supplement of “Bayesian reanalysis of null results reported in the New England Journal of Medicine: Strong yet variable evidence for the absence of treatment effects. This is a supplement of “Bayesian Reanalysis of Null Results Reported in the New England Journal of Medicine: Strong yet Variable Evidence for the Absence of Treatment Effects” by Hoekstra R, Monden R, van Ravenzwaaij D and Wagenmaker EJ. This document was written by Rei Monden (November, 2016). These plots were generated based on the 43 test statistics reported in the New England Journal of Medicine 2015.
(PDF)

S2 File All selected papers from the 2015 volume of the NEJM. (PDF)
