# Supplementary material for: Bayesian reanalysis of null results reported in medicine: Strong yet variable evidence for the absence of treatment effects
Source: PLoS One. 2018 Apr 25;13(4):e0195474. doi: 10.1371/journal.pone.0195474 (PMC5919013; doi:10.1371/journal.pone.0195474)
Supplement: S1 File — This is a supplement of “Bayesian Reanalysis of Null Results Reported in the New England Journal of Medicine: Strong yet Variable Evidence for the Absence of Treatment Effects” by Hoekstra R, Monden R, van Ravenzwaaij D and Wagenmaker EJ. This document was written by Rei Monden (November, 2016). These plots were generated based on the 43 test statistics reported in the New England Journal of Medicine 2015. (PDF) [file pone.0195474.s001.pdf]

Supplement of "Bayesian Reanalysis of Null Results Reported in the New England Journal of Medicine: Strong yet Variable Evidence for the Absence of Treatment Effects"

- This is a supplement of "Bayesian Reanalysis of Null Results Reported in the New England Journal of Medicine: Strong yet Variable Evidence for the Absence of Treatment Effects" by Hoekstra R, Monden R, van Ravenzwaaij D and Wagenmaker EJ. This document was written by Rei Monden (November, 2016).
- These plots were generated based on the 43 test statistics reported in the New England Journal of Medicine 2015.

Data

```
(nomisdata)
```

```
##    P.value    BF01 Total.N
## 1    0.420   6.190    3856
## 2    0.870  13.150     981
## 3    0.190  12.300     981
## 4    0.240  11.950    2033
## 5    0.470  68.540    6109
## 6    0.930 107.300    7945
## 7    0.620  89.650    5985
## 8    0.930  84.450    7768
## 9    0.700  18.860      86
## 10   0.360  11.470   10003
## 11   0.530   8.610     524
## 12   0.230  45.100   18446
## 13   0.480  85.340   10003
## 14   1.000  27.840    1044
## 15   0.900  77.690   10063
## 16   0.750   3.790     106
## 17   0.760  16.360    1053
## 18   0.190   9.940     549
## 19   0.160   2.530     260
## 20   0.970  10.870     517
## 21   0.370  17.090    2070
## 22   0.170   6.840     310
## 23   0.630  19.230    1832
## 24   0.890   2.660      33
## 25   0.450  40.210    7213
## 26   0.110  14.560    7213
## 27   0.800  11.040     791
## 28   0.260   7.320    1325
## 29   0.190   2.420     259
## 30   0.380  15.440     404
## 31   0.600  15.280    1612
## 32   0.720   7.440     404
## 33   0.660   9.140     369
## 34   1.000  18.310    2059
## 35   0.400  10.460    1523
```

```
## 36   0.220   3.130     252
## 37   0.190  12.440    1990
## 38   0.870  21.710    2287
## 39   0.500   9.050     826
## 40   0.930   5.610     162
## 41   0.820  44.240    6068
## 42   0.985   4.023     101
## 43   0.860 560.900    2447
```

Figure 2

```
par(cex.main = 1.1, mar = c(4.5, 6, 2, 9) + 0.1, mgp = c(3, 1, 0), cex.lab = 1.1,
    font.lab = 2, cex.axis = 1.1, las = 1)
plot(nomisdata$P.value, log(nomisdata$BF01), xlim = c(0, 1), ylim = c(log(1), log(1000)),
     xlab = "", ylab = "", cex.lab = 2, cex.axis = 2, las = 1, yaxt = "n", xaxt="n",cex=2,
     bty = "n", type = "p", pch = 21, bg = "grey")

labelsUpper = log(c(1000,300,100, 30, 10, 3, 1))

criticalP = c( 0, labelsUpper)
for (idx in 1:length(criticalP)) {
  abline(h = criticalP[idx], col = "darkgrey", lwd = 1, lty = 2)
}
abline(h = 0)
axis(side = 4, at = criticalP, tick = c(TRUE,FALSE,rep(TRUE,5)), las = 2,
     cex.axis = 1, labels = FALSE)
axis(side = 4, at = labelsUpper + 0.602, tick = FALSE, cex.axis = 1.2,
     labels = c("", "","Extreme", "Very strong", "Strong", "Moderate", "Anecdotal"))

axis(side = 2, at = c(criticalP), tick = TRUE, las = 2, cex.axis = 1.1,
     labels = c("1", "1,000","300","100", "30", "10", "3", ""))

mtext("Bayes factor in favor of the null hypothesis", side = 2.5, line = 3.2, las = 0, cex = 1.2)
grid::grid.text("Evidence", 0.97, 0.5, rot = 270, gp = grid::gpar(cex = 1.2))
mtext("P-values", side = 1, line = 2.5, las = 1, cex = 1.1)

axis(at=seq(0,1,by=0.1),side=1)
```

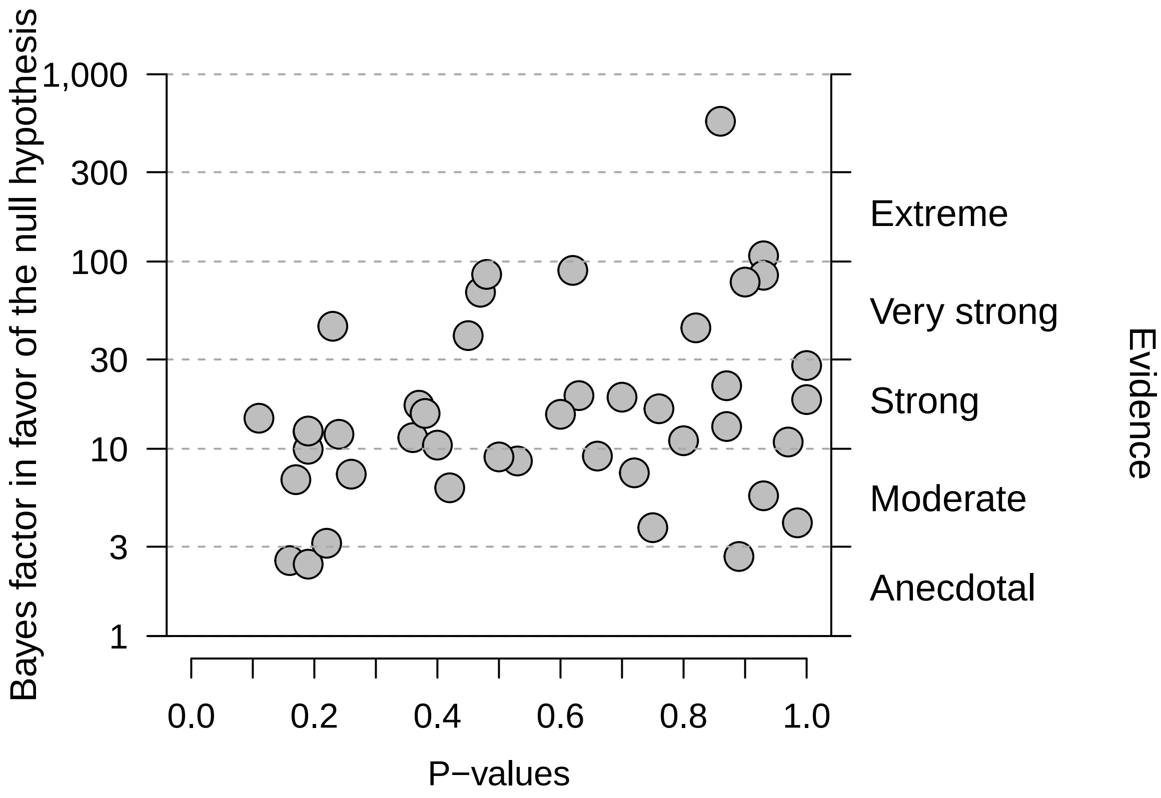

Figure 3

```
par(cex.main = 1.1, mar = c(4.5, 6, 2, 9) + 0.1, mgp = c(3, 1, 0), cex.lab = 1.1,
    font.lab = 2, cex.axis = 1.1, las = 1)
plot(log(nomisdata$Total.N), log(nomisdata$BF01), xlim = c(3, 11), ylim = c(log(1), log(1000)),
     xlab = "", ylab = "", cex.lab = 2, cex.axis = 2, las = 1, yaxt = "n", xaxt="n",cex=2,
     bty = "n", type = "p", pch = 21, bg = "grey")

labelsUpper = log(c(1000,300,100, 30, 10, 3, 1))

criticalP = c( 0, labelsUpper)
for (idx in 1:length(criticalP)) {
  abline(h = criticalP[idx], col = "darkgrey", lwd = 1, lty = 2)
}
abline(h = 0)
axis(side = 4, at = criticalP, tick = TRUE, las = 2, cex.axis = 1, labels = FALSE)
axis(side = 4, at = labelsUpper + 0.602, tick = FALSE, cex.axis = 1.2,
     labels = c("", "","Extreme","Very strong", "Strong", "Moderate", "Anecdotal"))

axis(side = 2, at = c(criticalP), tick = TRUE, las = 2, cex.axis = 1.1,
     labels = c("1","1,000","300", "100", "30", "10", "3", ""))

mtext("Bayes factor in favor of the null hypothesis", side = 2.5, line = 3.2, las = 0, cex = 1.2)
grid::grid.text("Evidence", 0.97, 0.5, rot = 270, gp = grid::gpar(cex = 1.2))
mtext("Total sample size", side = 1, line = 2.5, las = 1, cex = 1.1)
```

```
axis(at=c(log(30),log(100),log(300),log(1000),log(3000),log(10000),log(30000)),
     side=1,labels = c(30,100,300,"1,000","3,000","10,000","30,000"))
```

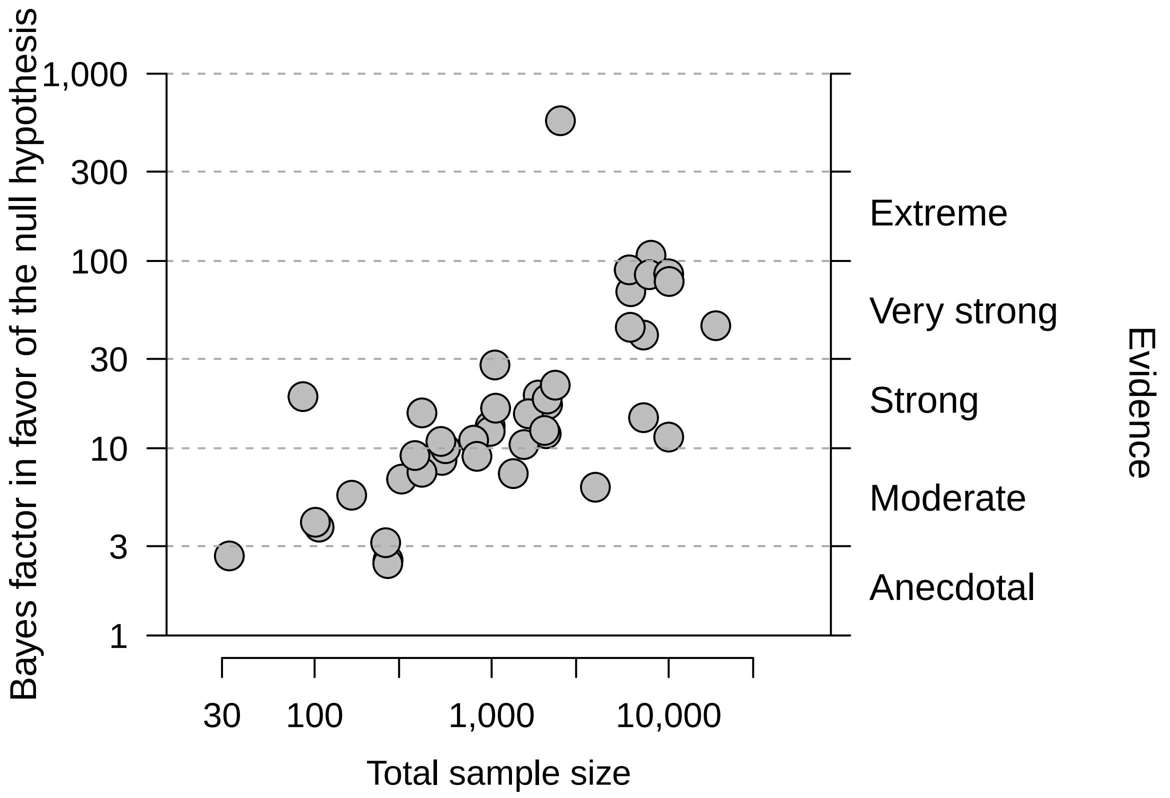

The correlation between Bayes factors and P-values:

```
cor(log(nomisdata$BF01),nomisdata$P.value)
```

```
## [1] 0.2896321
```

The correlation between Bayes factors and total sample sizes:

```
cor(log(nomisdata$BF01),log(nomisdata$Total.N))
```

```
## [1] 0.7198048
```
