# Supplementary material for: Bayesian reanalysis of null results reported in medicine: Strong yet variable evidence for the absence of treatment effects
Source: PLoS One. 2018 Apr 25;13(4):e0195474. doi: 10.1371/journal.pone.0195474 (PMC5919013; doi:10.1371/journal.pone.0195474)
Supplement: S2 File — (PDF) [file pone.0195474.s002.pdf]

All selected papers from the 2015 volume of the NEJM

No.	Issue	Page no.	URL	Reference in supplement *
2	372(1)	21-29	http://www.nejm.org/doi/full/10.1056/NEJMoa1404852	1
17	372(5)	407-417	http://www.nejm.org/doi/full/10.1056/NEJMoa1404595	2,3
20	372(5)	436-446	http://www.nejm.org/doi/full/10.1056/NEJMoa1412379	4
28	372(8)	711-723	http://www.nejm.org/doi/full/10.1056/NEJMoa1405044	5,6,7,8
39	372(10)	933-943	http://www.nejm.org/doi/full/10.1056/NEJMoa1404599	9
41	372(11)	997-1008	http://www.nejm.org/doi/full/10.1056/NEJMoa1403612	10
44	372(12)	1093-1103	http://www.nejm.org/doi/full/10.1056/NEJMoa1403789	11
50	372(13)	1213-1222	http://www.nejm.org/doi/full/10.1056/NEJMoa1412168	12
52	372(14)	1291-1300	http://www.nejm.org/doi/full/10.1056/NEJMoa1415516	13
55	372(14)	1324-1332	http://www.nejm.org/doi/full/10.1056/NEJMoa1414882	14
57	372(15)	1389-1398	http://www.nejm.org/doi/full/10.1056/NEJMoa1415098	15
58	372(15)	1399-1409	http://www.nejm.org/doi/full/10.1056/NEJMoa1500528	16
69	372(17)	1619-1628	http://www.nejm.org/doi/full/10.1056/NEJMoa1412278	17
78	372(19)	1812-1822	http://www.nejm.org/doi/full/10.1056/NEJMoa1408288	18
80	372(20)	1898-1908	http://www.nejm.org/doi/full/10.1056/NEJMoa1411480	19
84	372(21)	1996-2005	http://www.nejm.org/doi/full/10.1056/NEJMoa1411162	20
89	372(22)	2108-2117	http://www.nejm.org/doi/full/10.1056/NEJMoa1414293	21
90	372(23)	2185-2196	http://www.nejm.org/doi/full/10.1056/NEJMoa1503326	22
117	373(4)	307-316	http://www.nejm.org/doi/full/10.1056/NEJMoa1415340	23
135	373(8)	697-704	http://www.nejm.org/doi/full/10.1056/NEJMoa1506623	24
148	373(11)	997-1009	http://www.nejm.org/doi/full/10.1056/NEJMoa1507854	25,26
150	373(11)	1021-1031	http://www.nejm.org/doi/full/10.1056/NEJMoa1505489	27
153	373(12)	1095-1105	http://www.nejm.org/doi/full/10.1056/NEJMoa1506459	28
160	373(13)	1230-1240	http://www.nejm.org/doi/full/10.1056/NEJMoa1414827	29
161	373(14)	1295-1306	http://www.nejm.org/doi/full/10.1056/NEJMoa1507574	30
166	373(15)	1408-1417	http://www.nejm.org/doi/full/10.1056/NEJMoa1413534	31
170	373(16)	1507-1518	http://www.nejm.org/doi/full/10.1056/NEJMoa1504909	32, 33
171	373(16)	1519-1530	http://www.nejm.org/doi/full/10.1056/NEJMoa1500409	34, 35
182	373(19)	1824-1834	http://www.nejm.org/doi/full/10.1056/NEJMoa1508913	36
185	373(20)	1905-1915	http://www.nejm.org/doi/full/10.1056/NEJMoa1509038	37
188	373(20)	1937-1946	http://www.nejm.org/doi/full/10.1056/NEJMoa1505532	38
197	373(22)	2141-2148	http://www.nejm.org/doi/full/10.1056/NEJMoa1504927	39
200	373(23)	2225-2236	http://www.nejm.org/doi/full/10.1056/NEJMoa1415463	40
202	373(23)	2247-2257	http://www.nejm.org/doi/full/10.1056/NEJMoa1509225	41
206	373(24)	2336-2346	http://www.nejm.org/doi/full/10.1056/NEJMoa1508054	42
219	373(27)	2629-2641	http://www.nejm.org/doi/full/10.1056/NEJMoa1508502	43

* In the supplement file, all 43 results are ordered 1 to 43. In this column, it is indicated which results are related to which paper. It’s easy to see that one result was analyzed per paper, except for papers no. 17, 28, 148, 170 and 171.

Selection procedure

All articles published in the NEJM in 2015 : 219 papers - 12 (brief report) papers = 207 original articles

Selection of papers in which one of the following claims was made about a primary outcome:

- “no effect”,
- “small/negligible effect”,
- “no significant effect/difference”, or
- Simply reporting $P>.05$

This lead to a first selection of **45** articles (underscored were later taken out):

Article No. 2, 17, 20, 28, 39, 41, 44, 50, 52, 55,
57, 58, 69, 78, 80, 84, 89, 90, 117, 135,
148, 150, 153, 160, 161, 166, 170, 171, 182, 185,
188, 197, 200, 202, 206, 219,
60, 66, 67, 74, 174, 177, 199, 204, 212

↓

The following studies were excluded because no proportions were compared:

No. 60 (t-test)
66 (t-test),
74 (t-test),
199 (no proposition),
174 (t-test),
177 (non-inferiority test),
204 (general linear model),
212 (cox regression)

Paper 67 was excluded because our re-analysis showed a significant rather than a non-significant result

↓

⇒ Resulted in a final selection of **36** papers (**43** BFs calculated from the provided test statistics, given **7** extra results for primary outcomes within the selected papers).
